# Supplementary material for: Pyruvate Dehydrogenase Kinase 1 inhibition mediated oxidative phosphorylation enhancement in cartilage promotes osteoarthritis progression
Source: BMC Musculoskelet Disord. 2023 Jul 20;24:597. doi: 10.1186/s12891-023-06585-6 (PMC10357736; doi:10.1186/s12891-023-06585-6)
Supplement: Supplementary file 1 — Additional file 1. [file 12891_2023_6585_MOESM1_ESM.zip › BP.Gsea.1653623667859/GOBP_NEURONAL_STEM_CELL_POPULATION_MAINTENANCE.html]

Details for gene set GOBP\_NEURONAL\_STEM\_CELL\_POPULATION\_MAINTENANCE[GSEA]

|  || Dataset | 55457\_collapsed\_to\_symbols.cls.cls #OA\_versus\_normal.cls.cls #OA\_versus\_normal\_repos |
| Phenotype | cls.cls#OA\_versus\_normal\_repos |
| Upregulated in class | normal |
| GeneSet | GOBP\_NEURONAL\_STEM\_CELL\_POPULATION\_MAINTENANCE |
| Enrichment Score (ES) | -0.6466059 |
| Normalized Enrichment Score (NES) | -1.8957111 |
| Nominal p-value | 0.0 |
| FDR q-value | 0.05739133 |
| FWER p-Value | 0.733 |
Table: GSEA Results Summary

  

Fig 1: Enrichment plot: GOBP\_NEURONAL\_STEM\_CELL\_POPULATION\_MAINTENANCE      
 Profile of the Running ES Score & Positions of GeneSet Members on the Rank Ordered List

  

| SYMBOL | TITLE | RANK IN GENE LIST | RANK METRIC SCORE | RUNNING ES | CORE ENRICHMENT || 1 | SS18 | SS18 subunit of BAF chromatin remodeling complex [Source:HGNC Symbol;Acc:HGNC:11340] | 2677 | 0.282 | -0.1614 | No |
| 2 | CDH2 | cadherin 2 [Source:HGNC Symbol;Acc:HGNC:1759] | 2679 | 0.282 | -0.1151 | No |
| 3 | PRRX1 | paired related homeobox 1 [Source:HGNC Symbol;Acc:HGNC:9142] | 3657 | 0.189 | -0.1598 | No |
| 4 | PROX1 | prospero homeobox 1 [Source:HGNC Symbol;Acc:HGNC:9459] | 4299 | 0.128 | -0.1885 | No |
| 5 | FANCC | FA complementation group C [Source:HGNC Symbol;Acc:HGNC:3584] | 5138 | 0.061 | -0.2435 | No |
| 6 | ASPM | assembly factor for spindle microtubules [Source:HGNC Symbol;Acc:HGNC:19048] | 5164 | 0.059 | -0.2358 | No |
| 7 | PCM1 | pericentriolar material 1 [Source:HGNC Symbol;Acc:HGNC:8727] | 5900 | 0.002 | -0.2925 | No |
| 8 | FOXO1 | forkhead box O1 [Source:HGNC Symbol;Acc:HGNC:3819] | 6511 | -0.043 | -0.3328 | No |
| 9 | MMP24 | matrix metallopeptidase 24 [Source:HGNC Symbol;Acc:HGNC:7172] | 7767 | -0.139 | -0.4073 | No |
| 10 | MCPH1 | microcephalin 1 [Source:HGNC Symbol;Acc:HGNC:6954] | 10851 | -0.411 | -0.5791 | Yes |
| 11 | JAG1 | jagged canonical Notch ligand 1 [Source:HGNC Symbol;Acc:HGNC:6188] | 10977 | -0.427 | -0.5186 | Yes |
| 12 | FOXO3 | forkhead box O3 [Source:HGNC Symbol;Acc:HGNC:3821] | 11305 | -0.472 | -0.4665 | Yes |
| 13 | SRRT | "serrate, RNA effector molecule [Source:HGNC Symbol;Acc:HGNC:24101]" | 11626 | -0.521 | -0.4057 | Yes |
| 14 | REST | RE1 silencing transcription factor [Source:HGNC Symbol;Acc:HGNC:9966] | 12382 | -0.703 | -0.3489 | Yes |
| 15 | SOX2 | SRY-box transcription factor 2 [Source:HGNC Symbol;Acc:HGNC:11195] | 12485 | -0.742 | -0.2348 | Yes |
| 16 | NOTCH1 | notch receptor 1 [Source:HGNC Symbol;Acc:HGNC:7881] | 12555 | -0.776 | -0.1127 | Yes |
| 17 | HES1 | hes family bHLH transcription factor 1 [Source:HGNC Symbol;Acc:HGNC:5192] | 12676 | -0.849 | 0.0174 | Yes |
Table: GSEA details [plain text format]

  

Fig 2: GOBP\_NEURONAL\_STEM\_CELL\_POPULATION\_MAINTENANCE      
 Blue-Pink O' Gram in the Space of the Analyzed GeneSet

  

Fig 3: GOBP\_NEURONAL\_STEM\_CELL\_POPULATION\_MAINTENANCE: Random ES distribution      
 Gene set null distribution of ES for **GOBP\_NEURONAL\_STEM\_CELL\_POPULATION\_MAINTENANCE**
